# Effect of platform switching on the peri-implant bone: A finite element study

**DOI:** 10.4317/jced.52539

**Published:** 2015-10-01

**Authors:** Kheira Bouazza-Juanes, Amparo Martínez-González, Germán Peiró, Juan-José Ródenas, María-Victoria López-Mollá

**Affiliations:** 1 DDS. Assistant Professor. Department of Prosthodontics. Universidad Europea de Valencia. Spain; 2 MD, PhD. Associate Professor. Department of Prosthodontics. Universidad Europea de Valencia. Spain; 3 Graduate Student in Mechanical Engineer. Centro de Investigación en Ingeniería Mecánica. Universitat Politécnica de Valencia. Spain; 4 Eng, MSc, PhD. Associate Professor. Department of Mechanical and Materials Engineering. Centro de Investigación en Ingeniería Mecánica. Universitat Politécnica de Valencia. Spain; 5 DDS, PhD. Assistant Professor. Department of Prosthodontics. Universidad Europea de Valencia. Spain

## Abstract

**Background:**

There exists a relation between the presence and location of the micro-gap and the loss of peri implant bone. Several authors have shown that the treatments based on the use of platform switching result in less peri-implant bone loss and an increased tissue stability. The purpose of this study was to analyse the effect of the platform switching on the distribution of stresses on the peri-implant bone using the finite element method.

**Material and Methods:**

A realistic 3D full-mandible finite element model representing cortical bone and trabecular bone was used to study the distribution of the stress on the bone induced by an implant of diameter 4.1 mm. Two abutments were modelled. The first one, of diameter 4.1 mm, was used in the reference model to represent a conventional implant. The second one, of diameter 3.2 mm, was used to represent the implant with platform switching. Both models were subjected to axial and oblique masticatory loads.

**Results:**

The analyses showed that, although no relevant differences can be found for the trabecular bone, the use of platform switching reduces the maximum stress level in the cortical bone by almost 36% with axial loads and by 40% with oblique loads.

**Conclusions:**

The full 3D Finite Element model, that can be used to investigate the influence of other parameters (implant diameter, connexion type, …) on the biomechanical behaviour of the implant, showed that this stress reduction can be a biomechanical reasons to explain why the platform switching seems to reduce or eliminate crestal bone resorption after the prosthetic restoration.

** Key words:**Dental implant, platform switching, finite element method.

## Introduction

The success of the osseointegration of dental implants has been widely referenced in the literature, see for example (1,2). However, after osseointegration has taken place, it is frequent to observe a loss of bone around the neck of the implant once it is subjected to masticatory loading conditions (3). This bone loss takes place in the vicinity of the implant-abutment interface, space known as micro-gap, which is a confluence area of bone tissue and soft tissue (4).

According to many studies, there exists a relation between the presence and location of the micro-gap and the loss of peri-implant bone (4-7). These studies conclude that the conformation of a biological width similar but not equal to the biological width created the tooth, and next to the micro-gap, initiates a response that produces a significant bone loss in correlation with the proximity of the micro-gap to the crestal bone.

Some authors propose to modify the location of the implant-abutment interface by displacing the outer margin of the micro-gap towards the axis of the implant, moving it away from the bone crest (4). As a result, the horizontal component of the bone loss can be controlled or decreased, thus also decreasing the crestal bone loss (8). This was the origin of the platform switching (PS) or platform modification concept. The idea consists of using prosthetic components of smaller diameter than the platform of the implant. This will help to establish a horizontal biological seal and to keep the crestal bone away from the infiltration of inflammatory cells, which, as described by Ericsson *et al.* (9), occurs at the interface, thereby limiting the bone resorption.

Based on clinical studies, several authors have shown that the treatments based on the use of platform switching result in less peri-implant bone loss and an increased tissue stability (10,11).

However, it is still unclear if the good behaviour of the bone associated to the use of platform switching is only due to pure biological reasons, as biomechanics can also play an important role. A more appropriate distribution of the stresses on the bone or a more appropriate stress intensity, induced by the use of platform switching, could also be a reason behind the reduction of bone loss around the implant.

The objective of this work is to analyse the effect of the connection with platform switching on the distribution of stresses on the peri-implant bone.

## Material and Methods

The Finite Element Method (FEM) is a numerical analysis technique extensively used in the industry to evaluate, for example, the stress distribution in structural mechanical components. The use of the FEM has been rapidly increasing in the field of biomechanics during the last years. The FEM has been used in the analyses shown in this paper because of its ability to provide accurate representations of the stress distributions on the bone. The evaluation of these stress distributions through the current experimental analysis techniques would be unfeasible. The FEM has been already used to analyse the behaviour of implants, although, in most of them, substantial simplifications were considered to create the model of the mandible. As these simplifications can influence the results of the analysis, we decided to create a realistic 3D Finite Element model of the mandible that could later be used as a tool to analyse the influence other parameters on the biomechanical behaviour of the implant.

First, a realistic 3D surface CAD model of a human mandible used to host the implants was 

created from a 3D computed tomography processed with the program *SimPlant. 16* ® (*Materialise Dental*, NV, Leuven Belgium). This 3D surface was then used as the basis to create a CAD model of the volume of the mandible with SolidWorks (Solid Works 2015, SolidWorks Corp., Waltham, MA). The model was adequately adapted in the zone where the implant was going to be located to avoid complications during the Finite Element analysis run with the commercial software Ansys (ANSYS® Academic Research, Release 15.0, ANSYS, Inc, Canonsburg, PA, USA) once the model of the implant is included in the model. A model of an 11 mm long internal connection implant of diameter 4.1 mm was used in the analyses. Two models of abutments of diameters 4.1 mm and 3.2 mm were also created for the analyses. In the model, the bone tissue around the implant had approximate dimensions of 21x13 mm2 with 1.2 mm of cortical bone thickness.

As in other studies (12), the thread of the implant was modelled as a series of rings instead of modelling it as a helical thread. This approximation does not affect the quality of the results but considerably simplifies the model and reduces numerical issues during the Finite Element analysis. We assumed that the cortical and trabecular bones were rigidly bonded to the implant. Thus, we considered an optimal state of osseointegration.

We have analysed two models in our study ( Table 1), one of them, used as reference, corresponds to a conventional implant without PS, whereas the second one corresponds to an implant with PS.

**Table 1:**
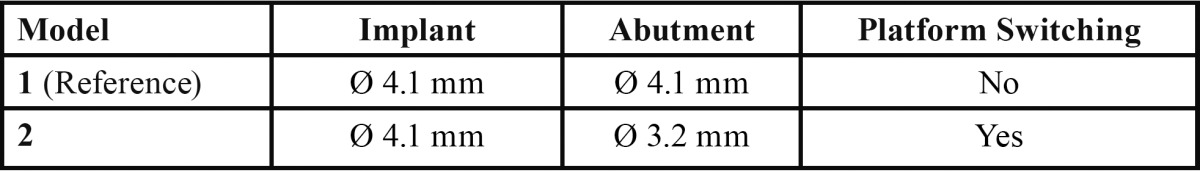
Models analysed with FEM.

A 100 N force was used in both models to simulate the masticatory loads. Two load cases were analysed. In the first case, we considered a vertical load aligned with the axis of the implant-abutment system. In the second case, the load was obliquely applied in the plane of the cross section of the mandible at an angle of 15° to the horizontal.

We assumed that homogeneous linear-elastic isotropic materials, as in other analyses shown in the bibliography (12,13). The properties of the materials, see  Table 2, were taken from references (12,13).

**Table 2:**
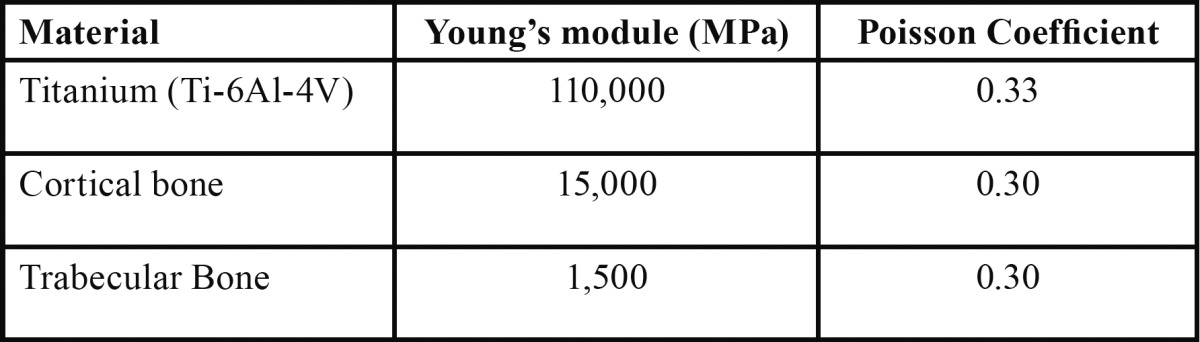
Material properties (12,13).

**Figure 1:**
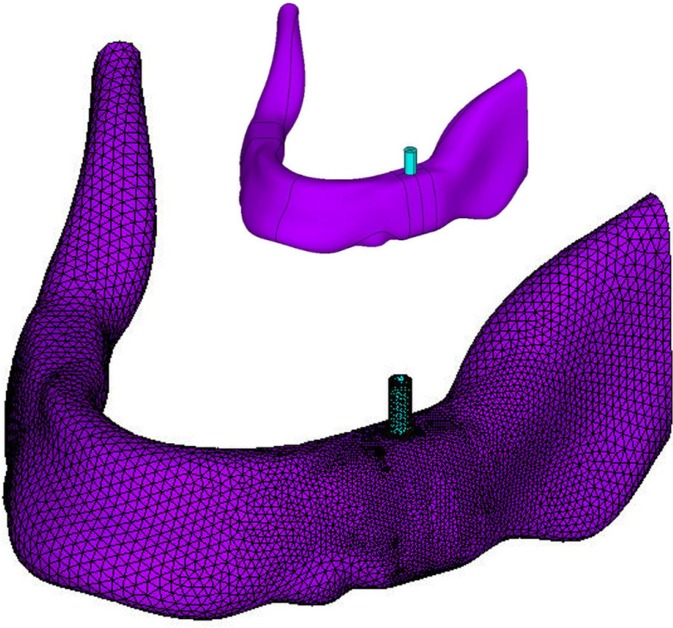
CAD and FEM models used for the analysis of the reference configuration (Model 1).

Figure 1 shows an example of the CAD and Finite Element models used for the analyses that, in this case, correspond to Model 1. As represented in the figure, the Finite Element model used small element sizes in the vicinity of the implant to have a detailed and accurate representation of the solution in this zone. Bigger element sizes were used in the rest of the model to reduce the computational cost of the analyses, but small enough as to maintain the required accuracy in the representation of the mandible.

## Results

**Figure 2:**
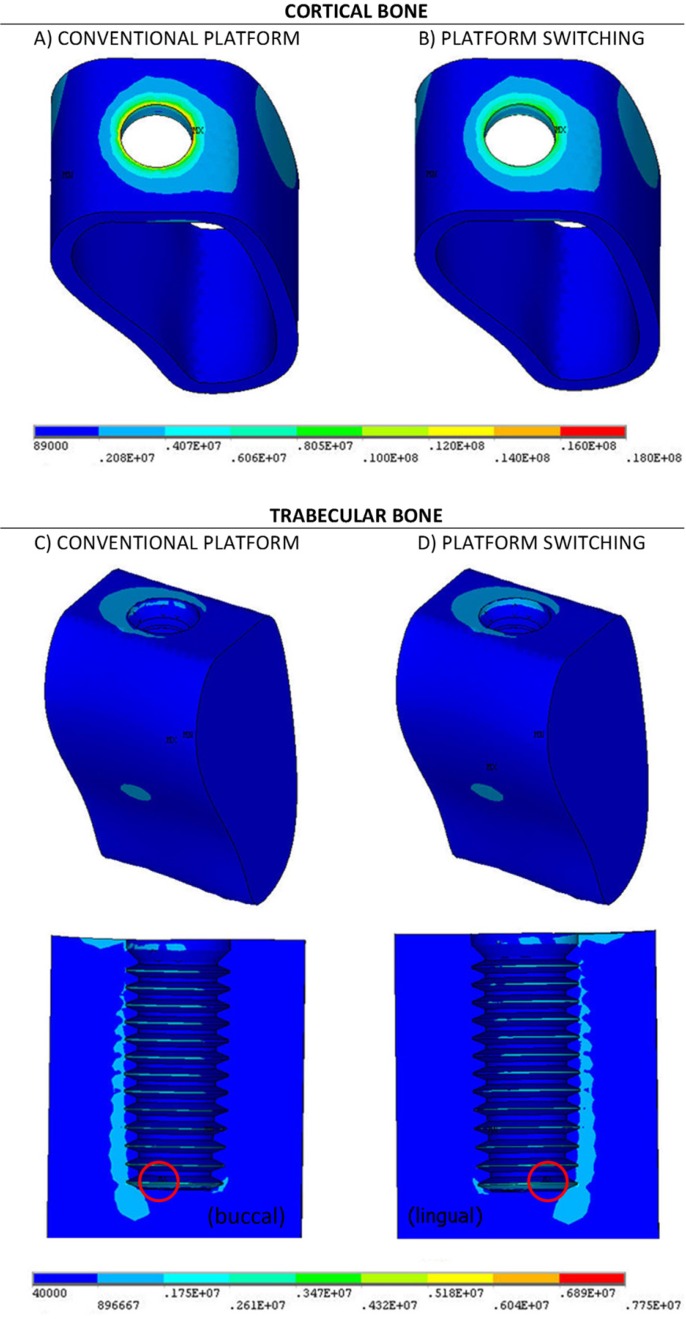
Von Mises (Pa) stress distribution on the bone under vertical load. A and B cortical bone. C and D trabecular bone.

**Figure 3:**
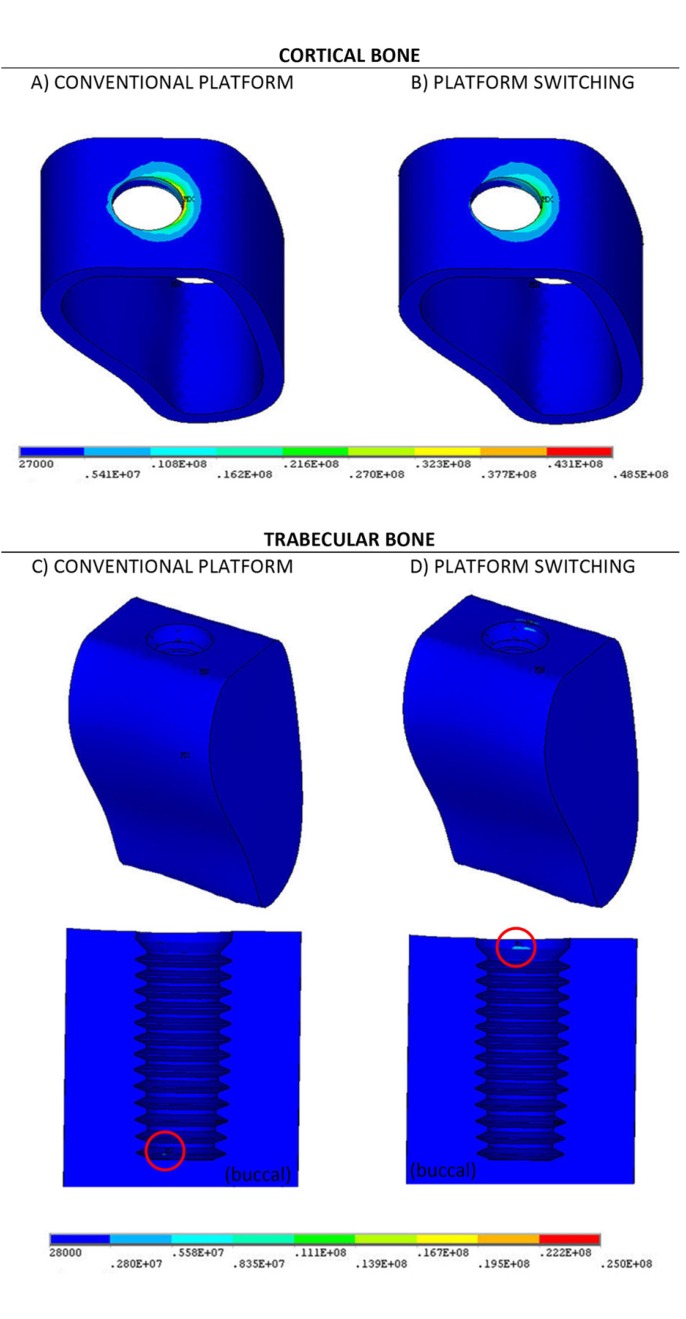
Von Mises (Pa) stress distribution on the bone under oblique load. A and B cortical bone. C and D trabecular bone.

The FEM provides the numerical values of the stresses at each point of the domain. The overall multiaxial stress state at each point is given by 6 stresses applied along different spatial directions. The von Mises stress, σvm, represents an uniaxial stress, equivalent to this multiaxial stress state. This magnitude has been used in this paper to represent the stress state at each point, as in many papers in the bibliography. The distribution of von Mises stresses will be graphically represented by colour maps (Figs. 2,3) where the cool colours (navy blue, light blue and turquoise blue) indicate low values of σvm, and the warm colours (yellow, orange and red) indicate the highest values.

We compared the maximum von Mises stress values obtained on the bone for the reference model (model 1, conventional implant without PS) with the results of model 2 (implant with PS). The results for the first load case (vertical load aligned with the axis of the implant) are represented in  Table 3.

**Table 3:**
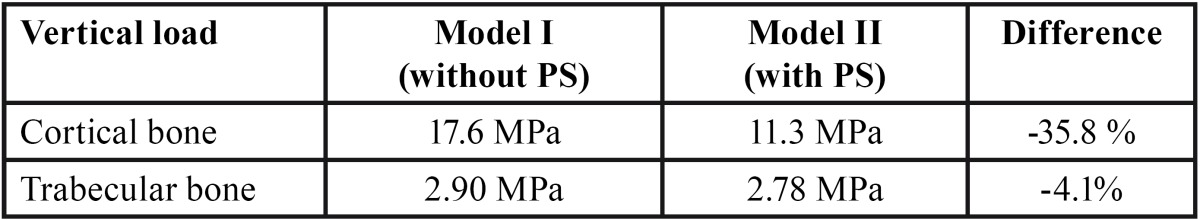
Effect of Platform Switching on the maximum von Mises stresses in bone under vertical load.

As shown in the  Table 3, in both cases, the maximum value of σvm in the cortical bone is higher than in the trabecular bone. The table clearly highlights the benefit of the use of platform switching on the maximum von Mises stress: σvm is significantly reduced by almost 36% in the cortical bone and only moderately by 4% in the trabecular bone. This last value cannot be considered a relevant result as it could be in the accuracy range of the stress values. This accuracy range is intrinsic to the numerical analysis: the numerical results are not the exact results due to a number of factors like the approximation inherent to the FEM, the simplifications of the geometry of the implant, the consideration of homogeneous isotropic material behaviour on the bone, etcetera.

As the load is aligned with the axis of the implant, in this case, the highest values of the von Mises stresses in the cortical bone are essentially concentric around the neck of the implant. The maximum value (represented by MX) is obtained at the top corner of the interface with the implant at the buccal region (Figs. 2A,B).

The intensity of the stresses in the trabecular bone (Figs. 2C,D) is clearly lower than in the cortical bone, where the stress only reach the lowest values of the scale. The highest values of the stresses are obtained around the thread and behind the implant. The maximum value of σvm, highlighted by a red circumference, is obtained on the lower thread, at the buccal region in the case of the conventional model (without PS) and at the lingual side in the case of PS.

 Table 4 compares the maximum values of σvm obtained in both models in the trabecular and cortical bones under the second load case, when the load is oblique. The stress distributions are represented in figure 3.

**Table 4:**
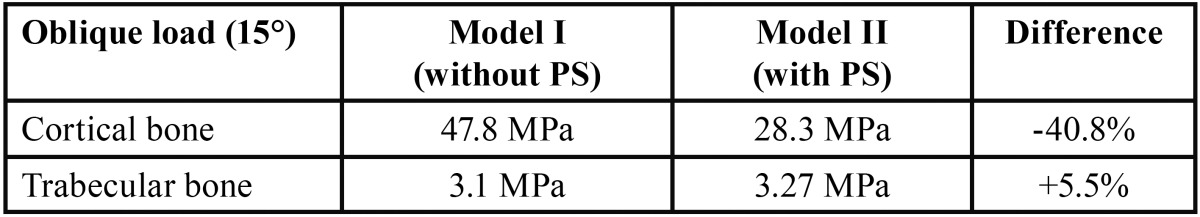
Effect of Platform Switching on the maximum von Mises stresses in bone under oblique load.

Comparing the results of  Table 3 and figure 2 with the results of  Table 4 and figure 3 we clearly observe that the oblique load produces, as expected, higher stress values than the vertical load, specially on the cortical bone, which required the use of different scale ranges in the figures.

With the oblique load, the highest values of σvm on the cortical bone are again obtained at the top corner of the interface with the implant. However, in this case these highest values are clearly located on the buccal side, because of the bending induced by the inclined load. We can clearly observe that in both models the maximum value was obtained at the same point, indicated by MX in the figure, although the use of PS reduced this value by a significant 41%. The results show that the use of PS increases the maximum value of σvm by 5.5% which, as in the previous case, cannot be considered as a relevant difference. In any case, the position of the maximum stress in the trabecular bone, indicated by red circumferences at the buccal side in figure 3 lies on the lower thread in the case reference model, and at the top of the site in the case of the model with PS.

## Discussion

Several studies, like this one, have shown the improvement in terms of a reduction in the stresses on the bone when configurations with platform switching are used in dental implants (13-15).

From the point of view of biomechanics, this reduction of the stress levels of the bone can be one of the reasons to explain why the platform switching seems to reduce or eliminate crestal bone resorption after the prosthetic restoration (15), as observed in various clinical studies (10,16). The lowest values of stress obtained in the peri-implant bone tissue when platform switching is used would lead to a reduction in the amount of micro-damage on the bone induced by the masticatory loads transmitted through the implant. Cappiello *et al.* (17) published a prospective study, based on the use of periapical X-rays, that considered 131 implants of which 75 were with PS. This study concluded that PS reduces the peri-implant crestal bone resorption and increases the long-term predictability of the treatment.

Most studies include the stresses distribution on the bone without distinguishing between trabecular or cortical bones (18). We have observed in this study that the use of platform switching does not significantly change the stress level on the trabecular bone whereas the cortical bone is highly influenced by its use.

In the finite element analysis presented in Maeda *et al.* (19) the authors show that the higher stress levels in the implant move towards the axis of the implant, i.e. away from the bone, in implants with platform switching. However, moving the higher stress levels towards the axis of the implant increases the stress on the screw. These authors conclude that this can lead to overloading and failure of the screw if the yield stress of the material is reached. Similar ideas have been also exposed by other authors. For example, Tabata *et al.* (15) observed an increased stress level on the screw and the implant when platform switching was used, although they showed that the stresses did not reach the yield limit of the titanium used for the implant. It is necessary to extend this kind of studies to confirm these findings as the platform switching could improve the bone response but it could also worsen the mechanical behaviour of the implant itself.

## Conclusions

The present study shows that platform switching reduces the maximum stress level on the cortical peri-implant bone with respect to the results obtained without platform switching. The stress reduction is obtained both with axial and oblique masticatory loads. This can represent a biomechanical explanation of the minimization of peri-implant bone resorption. The full 3D Finite Element model used in the analyses provided results in agreement with previous studies and will be used in future works to investigate the influence of other parameters (implant diameter, connection, …) on the biomechanical behaviour of the implant.
